# Preoperative nomogram for identifying invasive pulmonary adenocarcinoma in patients with pure ground-glass nodule: A multi-institutional study

**DOI:** 10.18632/oncotarget.11236

**Published:** 2016-08-11

**Authors:** Yunlang She, Lilan Zhao, Chenyang Dai, Yijiu Ren, Junyan Zha, Huikang Xie, Sen Jiang, Jingyun Shi, Shunbin Shi, Weirong Shi, Bing Yu, Gening Jiang, Ke Fei, Yongbing Chen, Chang Chen

**Affiliations:** ^1^ Department of Thoracic Surgery, Shanghai Pulmonary Hospital, Tongji University School of Medicine, Shanghai, P.R. China; ^2^ Department of Pathology, Shanghai Pulmonary Hospital, Tongji University School of Medicine, Shanghai, P.R. China; ^3^ Department of Radiology, Shanghai Pulmonary Hospital, Tongji University School of Medicine, Shanghai, P.R. China; ^4^ Department of Thoracic Surgery, The Affiliated Wujiang Hospital of Nantong University, Jiangsu, P.R. China; ^5^ Department of Thoracic Surgery, Nantong Sixth Peoples Hospital, Jiangsu, P.R. China; ^6^ Department of Thoracic Surgery, Fenghua Peoples Hospital, Zhejiang, P.R. China; ^7^ Department of Thoracic Surgery, The Second Affiliated Hospital of Soochow University, Jiangsu, P.R. China

**Keywords:** ground-glass nodule, lung adenocarcinoma, nomogram

## Abstract

**Purpose:**

To construct a preoperative nomogram to differentiate invasive pulmonary adenocarcinomas (IPAs) from preinvasive lesions in patients with solitary pure ground-glass nodules (GGN).

**Methods:**

A primary cohort of patients with pathologically confirmed pulmonary solitary pure GGN after surgery were retrospectively studied at five institutions from January 2009 to September 2015. Half of the patients were randomly selected and assigned to a model-development cohort, and the remaining patients were assigned to a validation cohort. A nomogram predicting the invasive extent of the solitary GGNs was constructed based on the independent risk factors. Predictive performance was evaluated by concordance index (C-index) and calibration curve.

**Results:**

Out of 898 cases included in the study, 501 (55.8%) were preinvasive lesions and 397 (44.2%) were IPAs. In the univariate analysis, lesion size (*p* < 0.001), lesion margin (*p* = 0.041), lesion shape (*p* < 0.001), mean computed tomography (CT) value (*p* = 0.018), presence of pleural indentation (*p* = 0.017), and smoking status (*p* = 0.014) were significantly associated with invasive extent. In multivariate analysis, lesion size (*p* < 0.001), lesion margin (*p* = 0.042), lesion shape (*p* < 0.001), mean CT value (*p* = 0.014), presence of pleural indentation (*p* = 0.026), and smoking status (*p* = 0.004) remained the predictive factors of invasive extent. A nomogram was developed and validation results showed a C-index of 0.94, demonstrating excellent concordance between predicted and observed results.

**Conclusions:**

We established and validated a novel nomogram that can identify IPAs from preinvasive lesions in patients with solitary pure GGN.

## INTRODUCTION

Pulmonary ground-glass nodules (GGN) have been increasingly encountered in routine clinical practice [1]. In general, two types of GGN include pure GGN and part-solid GGN. Part-solid GGNs contain both ground-glass opacity (GGO) and a solid component while pure GGNs are defined as those without solid components [2].

In terms of managing a solitary pure GGN, Fleischner Society recommends that lesions larger than 5 mm require a surveillance CT examination for a minimum of 3 years, if persistent and unchanged [3]. This is consistent with the American College of Chest Physicians recommendations [4]. According to the recently proposed IASLC/ATS/ERS classification [5], most of these lesions might be preinvasive lesions (atypical adenomatous hyperplasia, AAH; adenocarcinoma *in situ*, AIS). It is well established that these preinvasive lesions can be followed up alone or treated safely with limited resection, as they eventually might evolve into invasive lesion and require resection [6–8]. Meanwhile, the Japanese Society of CT Screening recommends that a workup should be performed to make a definitive diagnosis if a pure GGN is 15 mm or larger in maximal diameter on a thin-section computed tomography (TSCT) scan. Recently, several studies have demonstrated that pure GGNs of ≥ 10 mm in diameter is one of the TSCT scan features of invasive pulmonary adenocarcinoma (IPA; including minimally invasive adenocarcinoma, MIA and invasive adenocarcinoma, IA) [9, 10]. A nodule size > 16.4 mm in maximal diameter was associated with IA [11]. Thus, surgical resection should be considered for these larger pure GGN. It is important to discriminate IPAs from preinvasive lesions in patients with pure GGN before surgery, which could be helpful in selecting patients suitable for sublobar resection.

However, to date, only maximal diameter of lesion has been used to assess the nodules. Hence, we aimed to construct a preoperative nomogram with several independent predictive factors to differentiate IPAs from preinvasive lesions in patients with solitary pure GGNs.

## RESULTS

### Baseline characteristics

The characteristics of the model-development and validation cohorts are summarized in Table 1. The study included 898 patients with solitary pure GGN. Regarding gender, 311 (34.6%) were men and 587 (65.4%) were women, with the mean age of 54.1 ± 11.0 years. The pathological status indicated 501 (55.8%) preinvasive lesions and 397 (44.2%) IPAs.

**Table 1:** Basal characteristics*

Characteristics		Overall cohort (*n* = 898)	Derivation Cohort (*n* = 449)	Validation Cohort (*n* = 449)	*P* Value
*Host factors*					
Age, yr		54.12±11.00	54.19±11.67	54.05±10.31	0.856
Sex (male:female)		311(34.6):587 (65.4)	164 (36.5): 285 (63.5)	147 (32.7): 302 (67.3)	0.233
Family tumor history					
	With	24(2.7)	12(2.7)	12(2.7)	1.000
	Without	874(97.3)	437(97.3)	437(97.3)	
Presence of symptoms					
	Present	175(19.5)	79(17.6)	96 (21.4)	0.152
	Absent	723 (80.5)	370(82.4)	353 (78.6)	
Smoking status					
	Never smoker	804(89.5)	398 (88.6)	406(90.4)	0.383
	Current or former smoker	94(10.5)	51 (11.4)	43(9.6)	
CEA, ng/mL		1.52±1.27	1.60±1.29	1.45±1.24	0.389
*Radiologic factors*					
Lesion size (cm)		1.22±0.72	1.20±0.53	1.23±0.87	0.526
Lesion shape					
	Irregular	430 (47.9)	222 (49.4)	208 (46.3)	0.618
	Oval	228(25.4)	109 (24.3)	119 (26.5)	
	Round	240 (26.7)	118 (26.3)	122 (27.2)	
Lesion margin					
	Easily differentiated	536 (59.7)	256 (57.0)	280 (62.4)	0.103
	Uneasily differentiated	362 (40.3)	193 (43.0)	169 (37.6)	
Pleural indentation					
	Present	242(26.9)	118 (25.4)	128(28.5)	0.292
	Absent	656(73.1)	335(74.6)	321(71.5)	
Mean CT value, HU		-565.31±114.80	-567.55±117.60	-563.07±112.01	0.559
Lesion location					
	Left upper lobe	252(28.1)	131(29.2)	121(26.9)	0.316
	Left lower lobe	99(11.0)	45(10.0)	54(12.0)	
	Right upper lobe	353(39.4)	174(38.8)	180(40.1)	
	Right middle lobe	58(6.5)	24(5.3)	34(7.6)	
	Right lower lobe	135(15.0)	75(16.7)	60(13.4)	
Bubble sign					
	Present	163 (18.2)	84 (18.7)	79 (17.6)	0.665
	Absent	735 (81.8)	365 (81.3)	370 (82.4)	
Air bronchogram					
	Present	316(35.2)	166(37.0)	150(33.4)	0.264
	Absent	582(64.8)	283(63.0)	299(66.0)	
Vessel through					
	Present	494 (55.0)	246 (54.8)	248 (55.2)	0.893
	Absent	404 (45.0)	203 (45.2)	201 (44.8)	
*Pathologic factors*					
Pre-invasive lesion					
	AAH	128(25.5)	59(24.4)	69(26.6)	0.562
	AIS	373(74.5)	183(75.6)	190(73.4)	
Invasive pulmonary adenocarcinoma					
	MIA	192(48.4)	93(44.9)	99(52.1)	0.153
	IA	205(51.6)	114(55.1)	91(47.9)	

* Values are presented as no. (%) or mean ± SD.

AAH, atypical adenomatous hyperplasia; AIS, adenocarcinoma in situ; MIA, minimal invasive adenocarcinoma; IA, invasive adenocarcinoma.

### Univariate and multivariate analysis

In the derivation cohort, mean CT values were -602.42±99.93 HU in the preinvasive adenocarcinoma group and -526.80±123.69 HU in the IPA group. The maximum sensitivity and specificity were obtained at a cutoff value of −560.20 HU by receiver operating characteristics curve analysis. Mean CT values were changed to categorical variables, that is, more than or equal to −560.20 HU or less than −560.20 HU.

According to the univariate analysis in Table 2, lesion size (*p* < 0.001), lesion margin (*p* = 0.041), lesion shape (*p* < 0.001), mean CT value (*p* = 0.018), presence of pleural indentation (*p* = 0.017), and smoking status (*p* = 0.014) were significantly associated with invasive extent. In multivariate analysis, lesion size (*p* < 0.001), lesion margin (*p* = 0.042), lesion shape (*p* < 0.001), mean CT value (*p* = 0.014), presence of pleural indentation (*p* = 0.026), and smoking status (*p* = 0.004) still had statistically significant effect on differentiating IPAs from preinvasive lesions (Table 3).

**Table 2:** Univariate logistic regression analysis of the association between clinicoradiological factors and pathologic status

Factor		Odds Ratio (95% CI)	*p* Value
Age		1.00(0.97-1.04)	0.953
Sex			
	Female	Reference	
	Male	0.92(0.38-2.27)	0.428
Family tumor history			
	With	Reference	
	Without	0.73 (0.07-7.73)	0.791
Presence of symptoms			
	Present	Reference	
	Absent	0.34(0.12-0.96)	0.085
Smoking status			
	Current or past smoker	Reference	
	Non-smoker	0.19 (0.05-0.71)	0.014
CEA, ng/mL		1.18 (0.62 - 1.35)	0.814
Tumor diameter, cm		21.23 (5.95-75.77)	<0.001
Pleural indentation			
	Present	Reference	
	Absent	0.23 (0.07-0.77)	0.017
Mean CT value, HU			
	<-560.20	Reference	
	≥-560.20	2.80 (1.20-6.55)	0.018
Tumor location			
	Left upper lobe	Reference	0.520
	Left lower lobe	0.93 (0.25-3.53)	0.919
	Right upper lobe	1.40 (0.51-3.84)	0.518
	Right middle lobe	0.38 (0.06-2.34)	0.297
	Right lower lobe	0.60 (0.18-2.03)	0.410
Lesion margin			
	Uneasily differentiated	Reference	
	Easily differentiated	0.47(0.20-1.11)	0.041
Lesion shape			
	Irregular	Reference	<0.001
	Oval	0.09(0.04-0.25)	<0.001
	Round	0.02(0.01-0.09)	<0.001
Bubble sign			0.856
	Present	Reference	
	Absent	1.12(0.33-3.81)	
Air bronchogram			
	Present	Reference	
	Absent	0.52(0.20-1.31)	0.162
Vessel through			
	Present	Reference	
	Absent	1.29(0.54-3.07)	0.568

CI, confidence interval; OR, odds ratio.

**Table 3:** Multivariate logistic regression analysis of the association between clinicoradiological factors and pathologic status

Factor		Odds Ratio (95% CI)	*p* Value
Tumor diameter, cm		33.36(10.21-109.02)	<0.001
Smoking status			
	Current or past smoker	Reference	
	Non-smoker	0.17(0.05-0.56)	0.004
Lesion margin			
	Uneasily differentiated	Reference	
	Easily differentiated	0.43 (0.19 - 0.97)	0.042
Lesion shape			
	Irregular	Reference	<0.001
	Oval	0.09 (0.04-0.21)	<0.001
	Round	0.03(0.01-0.10)	<0.001
Pleural indentation			0.026
	Present	Reference	
	Absent	0.31 (0.11 - 0.87)	
Mean CT value, HU			0.014
	<-560.20	Reference	
	≥-560.20	2.58(1.21-5.47)	

CI, confidence interval; OR, odds ratio.

### Construction and validation of the nomogram

**Figure 1:**
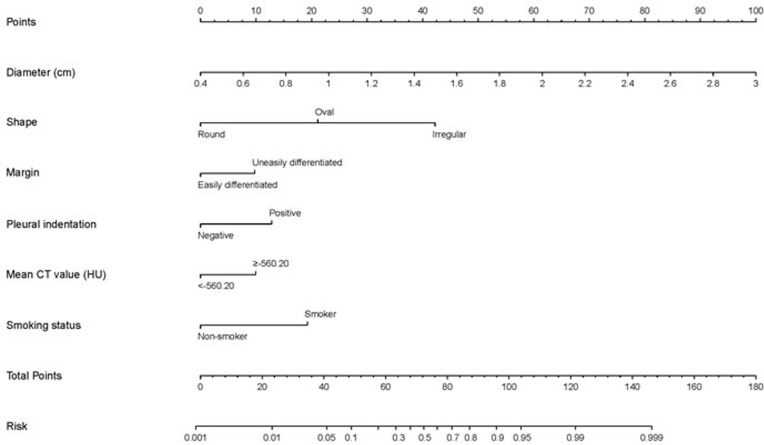
Nomogram for predicting probability of invasive status To obtain the nomogram-predicted probability locate the patient values at each axis, and draw a vertical line to the “Points” axis to determine the number of points attributed to each variable value, determine total number of points for all variables, and locate the sum on the “Total Points” line to assess the individual probability of invasive status.

A nomogram for differentiating IPAs from preinvasive lesions in patients with solitary pure GGNs based on the results of the multivariate analysis is shown in Figure 1. The model is expressed as follows:

(1) Probability of IPA = e^x^ / (1 + e^x^), where e is the base of natural logarithms.

(2) x = -3.1957 + (3.5953*Lesion size [cm]) - (1.9740*Lesion shape) + (0.9130*Lesion margin) + (1.1996*Pleural indentation) + (0.9270*Mean CT value [HU]) + (1.7979*Smoking status)

In the equation, lesion shape is scored as 1 for irregular, 2 for oval, and 3 for round; lesion margin is scored as 1 for uneasily differentiated and 0 for easily; pleural indentation is scored as 1 if present and 0 if not; mean CT value is scored as 0 for less than -560.20HU and 1 for more than or equal to -560.20HU; and smoking status is scored as 1 for a smoker or ever a smoker and 0 for non-smoker.

**Figure 2:**
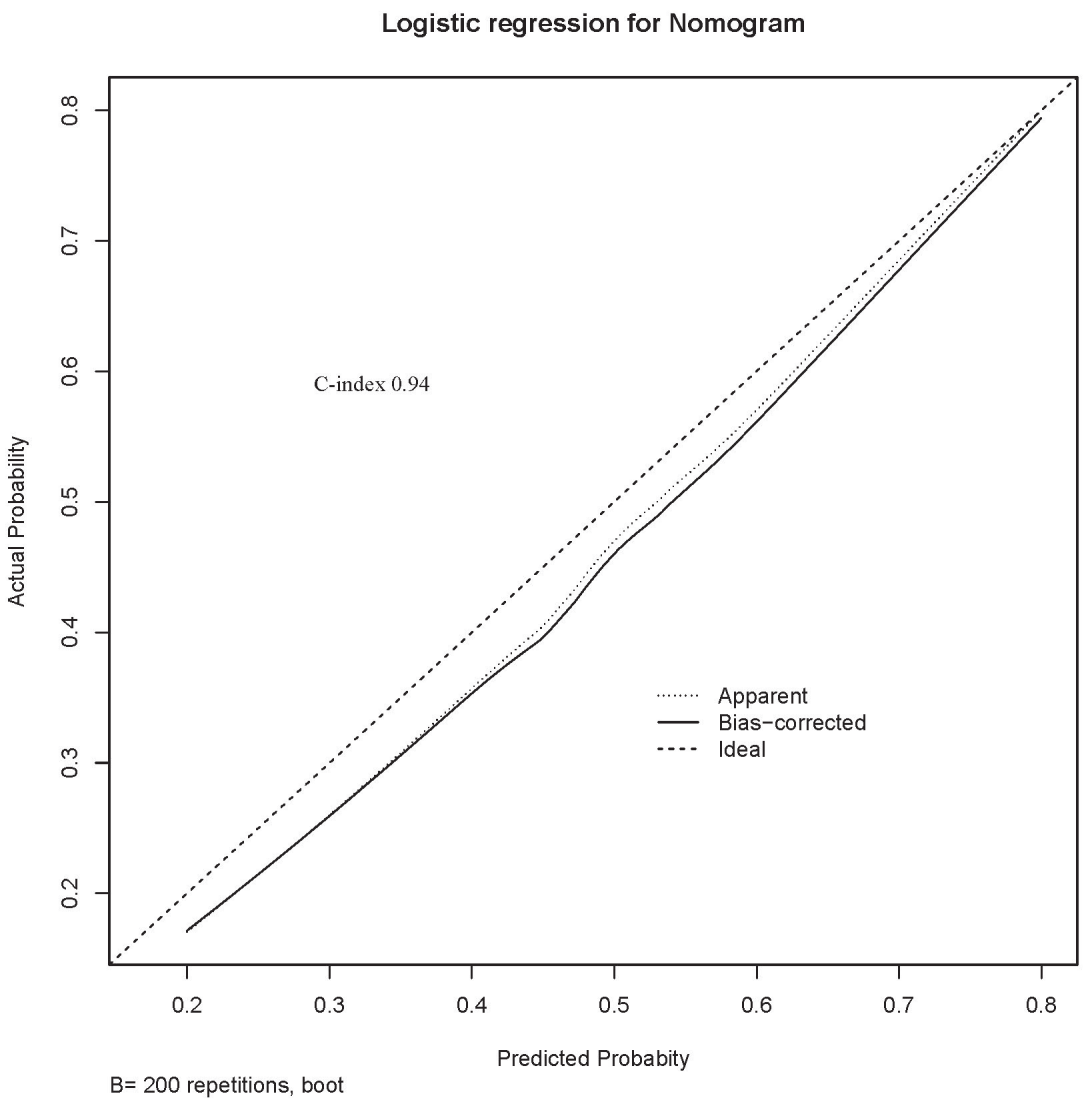
Calibration plot of relationship of predicted and actual probabilities The x-axis shows the prediction calculated using the nomogram, and the y-axis gives observed rates of invasive status. The dashed line indicates a reference line, where an ideal nomogram would lie. The solid line indicates performance of the nomogram applied to the validation cohort. The solid line is close to the dashed line of the ideal nomogram with a C-index of 0.94.

A calibration curve validating model performance is shown in Figure 2. The performance of our nomogram is plotted as a solid line. The nomogram calibration plot demonstrated virtually ideal predictions. The rate of predicted invasive status paralleled the observed rate of invasive status, nearly corresponding to the 45° line. The C-index of validation model was 0.94, indicating a good discrimination. The correspondence seen between actual and ideal nomogram predictions suggests good calibration of the nomogram in the validation cohort.

## DISCUSSION

The major findings of this study are as follows. (1) Lesion size, lesion shape, lesion margin, the mean CT value, the presence of plural indentation, and the smoking status were specific discriminators of IPA from preinvasive lesions. (2) A nomogram to differentiate IPAs from preinvasive lesions was developed, and validation results showed a C-index of 0.94 that demonstrated excellent concordance between predicted and observed results.

Nowadays, the resection criteria of GGNs were still unresolved, although several important guidelines were published in 2011 and 2013 [3, 4, 9]. Before the proposed IASLC/ATS/ERS classification in 2011 [5], some studies [12, 13] showed that 8 mm was the optimal cut-off value for distinguishing benign from malignant nodules. In our institutions, a significant increase in size (over 2 mm) may also indicate resection; therefore, we performed surgical resections on some patients with GGN smaller than 10 mm. Therefore, the mean size of GGNs in our cohort was 12.2 mm, which is smaller compared to the recommended resection diameter of 15 mm. In the current study, the rate of IPAs (MIA and IA) was 44.2% (401/898) in pure GGNs; however, it is comparable with previous reports ranging from 34% (14/41) [10] to 78% (73/93) [14]. Considering that we enrolled large sample of patients at different centers from 2009 to 2015, our data is comprehensive and convincing.

We confirmed that larger lesion size and higher mean CT value were associated with IPA. Previous studies by Lee at al. [10], Lim et al. [11], and Kitami et al. [15] also demonstrated that lesion size and the attenuation value of GGN were specific discriminators of IPAs from preinvasive lesions. Increased attenuation within GGN in IPAs is believed to reflect thickened myofibroblastic stroma caused by the infiltration of invasive tumor cells [16, 17].

In the current study, lesion shape, lesion margin, and the presence of pleural indentation were also shown to discriminate IPAs from pre-invasive lesions. Lee et al. found that lobulated margin was more frequent in IPAs compared to pre-invasive lesions [12], but it was not shown to be a significant factor in multivariate analysis. Considering the results of Jin et al. [14], which indicated significantly different margin among preinvasive lesions and IPAs (*p* = 0.02), it may be reasonable to infer that irregular GGNs with ill-defined margin have a higher probability of IPAs. Meanwhile, central fibrosis and resultant tissue contraction could cause fibrotic strands around the tumor, which were recognized on CT as pleural indentations. Furthermore, the foci of fibrosis would become larger with the increased invasive grade of tumor. This mechanism suggests that pleural indentation is significantly associated with invasive extent., which is consistent with the findings of Takashima et al. [18] and Liang et al. [19] on mixed GGN.

Many studies have reported a correlation between smoking status and malignancy. Based on a survey of 67 patients with pulmonary nodules with GGO, Kobayashi et al. [20] reported that smoking history was associated with GGN growth. Notably, Chang et al. [21] analyzed 122 GGNs from patients with no history of malignancy, finding a higher proportion of smokers in the growth group. Nodule growth is important for differentiating those who are malignant from those who should be followed for future growth. Thus, GGN growth has been found to be associated with IPAs while smoking status has been found to be a significant factor for differentiating IPAs from preinvasive lesions.

A nomogram is a graphical statistical tool that uses different variables to calculate potential risk. Nomograms provide estimates based on the specific characteristics of an individual patient. The nomogram in our study showed high prediction capability in the validation cohort, as the calibration plot revealed a similar distribution to the ideal reference line. In a similar study performed by Lee et al. [10], they found that lesion size was the sole predictor of invasive extent. Moreover, Liang et al. [19] demonstrated that the amount of blood vessels (*n* ≥ 1) was also an independent risk factor able to differentiate IPAs from preinvasive lesions. Based on these results, we incorporated more variables compared to previous prediction models. Further, we believe that the established nomogram represents a more precise and easy to use the scoring system. The most important benefit of the nomogram is that risk can be assessed using noninvasive procedures before the surgery. Identifying subgroups of patients at different risk for invasive extent might have a positive effect on the treatment or care options. In addition, this tool could provide information on patient stratification in the design of clinical studies, improving equivalence between study arms.

This study has several limitations. First, this was a retrospective study that included only pathologically confirmed GGNs, which means that it is subjected to potential bias. Accordingly, our results should be further validated in a prospective manner. Second, we used several types of CT scanners with different detector numbers and different section thicknesses. Nodules scanned with a 2.5-mm-section thickness were also included. Recently published Fleischner Society guidelines for GGN evaluation recommend using a TSCT scan (1.0-mm-section thickness) technique. Therefore, nodule selection and the potential inclusion of nodules had some degree of soft-tissue attenuation. Third, the CT features in this study were derived from the visual estimations by radiologists; thus, they can be significantly influenced by subjective or bias on the part of the observers. However, visual estimation is a current reference standard for lesion estimation, and it is not technically easy to estimate automatically.

In conclusion, we found that smoking status and radiologic characteristics (lesion diameter, shape, margin, pleural indentation, and mean CT value) were specific discriminators of IPAs in pure GGNs. Our study constitutes the first nomogram to accurately discriminate IPAs from preinvasive lesions in patients with solitary pure GGNs. This model could assist surgeons and patients in clinical decision-making and treatment tailoring.

## MATERIALS AND METHODS

### Patient population

This retrospective study included eligible patients with pulmonary solitary pure GGN pathologically confirmed after surgery between January 2009 and September 2015 from the departments of cardiothoracic surgery of five institutions in China (Shanghai pulmonary hospital of Tongji university, Shanghai; The Second Affiliated Hospital of Suzhou University, Jiangsu; The Affiliated Wujiang Hospital of Nantong University, Jiangsu; Nantong Sixth People's Hospital, Jiangsu; Fenghua People's Hospital, Zhejiang). Ethical approval was obtained from participating institutions.

We searched the CT scan reports using the keywords “GGO,” “GGN,” “non-solid nodule,” “part-solid nodule,” “ground-glass opacity,” or “ground-glass nodule.” Subsequently, two thoracic radiologists (Dr. Sen Jiang and Dr. Jingyun Shi) who were blinded to the pathologic results evaluated the CT scans and assessed the nodules for inclusion. Pure GGNs were defined as those comprising only ground glass opacity (GGO) on lung window images (window width, 1200 Hounsfield units [HU]; window level, -500 HU) with no or barely identifiable soft-tissue attenuation within the lesion on the mediastinal window images (window width, 450 HU; window level, 50 HU) at TSCT. Our inclusion criteria were as follows: (1) solitary pure GGN; (2) lesion size less than 3 cm; (3) no tumor history; and (4) no distant metastasis.

### Surgical resection and histological evaluation

All patients had definite diagnoses based on surgical resection. The resection criteria were as follows: (1) In pure GGNs < 10 mm, surgical resection should be considered if there is an increase in size ≥ 2 mm during 6 months of follow-up. (2) In pure GGNs ≥ 10 mm without significant changes in the initial 3 months of follow-up, we recommended surgical excision for GGNs ≥ 15 mm, whereas we recommended chest CT follow-up for one year or surgical excision for GGNs measuring 10-15 mm in size [22]. Each surgical specimen (entire tumor) was formalin fixed and stained with haematoxylin-eosin in accordance with routine regulations of the five hospitals, which were reviewed according to the new IASLC/ATS/ERS classification criteria, and tumor histologic subtypes were recorded. All GGNs were divided into two groups, a preinvasive lesion group (AAH, AIS) and an IPA group (MIA, IA).

### Clinical data collection and CT imaging evaluation

The following clinical data were collected: age, sex, presence of symptoms, smoking status, family tumor history, the level of carcinoembryonic antigen (CEA). Chest CT was performed using the following scanners: Somatom Definition AS (Siemens Medical Systems, Germany); Brilliance (Philips Medical Systems, Netherlands); and Lightspeed Ultra (GE Medical Systems, Milwaukee, Wis) with 120 kVp, 100-200 mAs, pitch of 0.875-1.5, and collimation of 1-2.5 mm. Images were reconstructed using a medium sharp reconstruction algorithm with a thickness of 1-2.5 mm. CT scans were obtained from all patients in the supine position at full inspiration. All chest CT scans were reviewed for the following information: lesion location, lesion size (the maximal diameter), lesion shape (round, oval, or irregular), lesion margin (easily differentiated: smooth; uneasily differentiated: spiculated, lobulated, or both), presence of bubble lucency, pleural indentation, air bronchogram or vessels through, and the mean CT value.

Lesion size was defined as the longest tumor diameter on the transverse lung window image. Any shape that was not round or oval was defined as irregular. A smooth margin was defined when there were neither ins-and-outs (lobes) nor spiculation in the lesion surface. Lobulated margin was defined when a portion of the lesion's surface showed a wavy or scalloped configuration. Spiculated margin was defined as the presence of strands extending from the nodule margin into the lung parenchyma without reaching the pleural surface. Bubble lucency was considered present when small spots of round or ovoid air attenuation were present within the GGN. A pleural indentation was defined as a linear attenuation heading toward the pleura or the major or minor fissure from a GGN [2, 11, 12]. An air bronchogram was considered present when air-filled bronchi were seen within a GGN. The mean CT attenuation value (HU) was measured by placing a region of interest (ROI) of 15 mm^2^ in three different sites within the nodule, barring the vessels and bronchioles [14].

### Statistical analysis

Half of the patients were randomly selected and assigned to a model-development cohort, and the other half were assigned to a validation cohort. Nominal categorical variables were compared using χ^2^ tests, and ordinal categorical variables were compared using Wilcoxon rank-sum tests. Univariate logistic regression analysis was used to determine an association between the invasive extent of pure GGN and several clinical and imaging variables. Factors with a *p* value less than 0.05 in univariate analysis were included in the multivariate logistic regression model. The multivariate logistic regression models determined the effects of multiple factors on a nomogram, and only the factors with a p-value less than 0.05 were incorporated into the nomogram [23]. In validation cohort, the performance of the nomogram is assessed using the concordance index (C-index). The C-index was used to estimate the probability of concordance between predicted and observed responses. The C-index ranges from 0.5 to 1.0, with a value of 0.5 indicating a random chance and 1.0 indicating a perfect ability to correctly discriminate the outcome with the model. All analyses were performed using the SPSS version 19.0 (SPSS, Chicago, IL, USA) and R version 3.2.2 (The R Foundation for Statistical Computing, Vanderbilt University, Nashville, TN). A *p*-value < 0.05 was considered significant.
